# 2-(4-Chloro­phen­yl)-1-pentyl-4,5-di­phenyl-1*H*-imidazole

**DOI:** 10.1107/S1600536813018229

**Published:** 2013-07-13

**Authors:** Shaaban K. Mohamed, Mehmet Akkurt, Kuldip Singh, Adel A. Marzouk, Antar A. Abdelhamid

**Affiliations:** a Chemistry and Environmental Division, Manchester Metropolitan University, Manchester, M1 5GD, England; b Chemistry Department, Faculty of Science, Mini University, 61519 El-Minia, Egypt; c Department of Physics, Faculty of Sciences, Erciyes University, 38039 Kayseri, Turkey; d Department of Chemistry, University of Leicester, Leicester, England; e Pharmaceutical Chemistry Department, Faculty of Pharmacy, Al Azhar University, Egypt; f Chemistry Department, Faculty of Science, Sohag University, 82524 Sohag, Egypt

## Abstract

In the title compound, C_26_H_25_ClN_2_, the phenyl rings and the 2-(4-chloro­phen­yl) group make dihedral angles of 30.03 (11), 67.49 (12) and 41.56 (11)°, respectively, with the imidazole ring. In the crystal, the mol­ecules inter­act with each other *via* very weak C—H⋯π contacts, forming layers parallel to (110).

## Related literature

For biological applications of imidazole derivatives, see: Shalini *et al.* (2011 ▶); Ramesh *et al.* (2012 ▶); Wolkenberg *et al.* (2004 ▶). For related structures, see: Simpson *et al.* (2013 ▶); Akkurt *et al.* (2013 ▶).
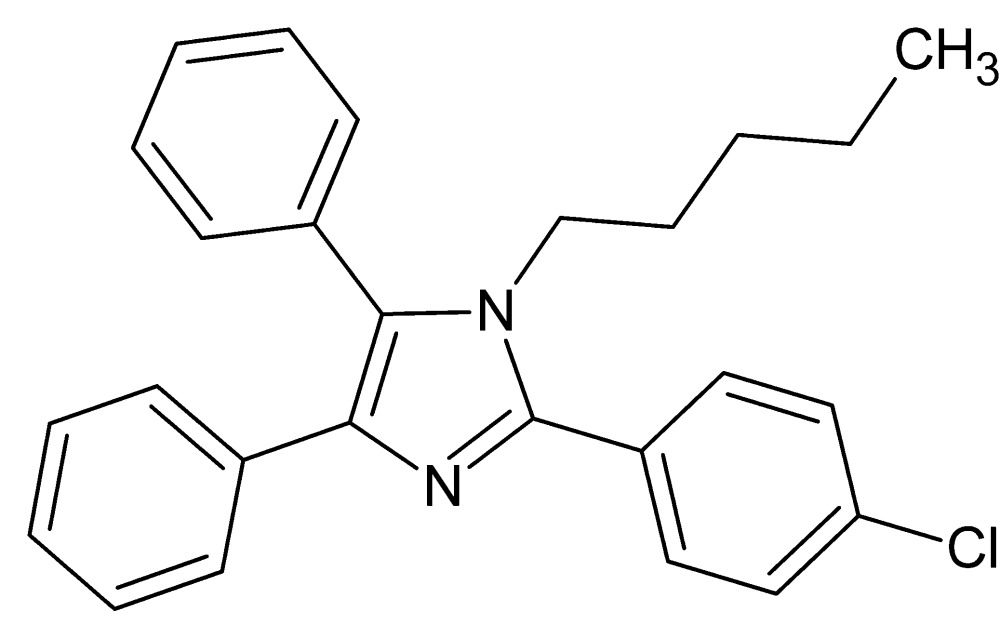


## Experimental

### 

#### Crystal data

C_26_H_25_ClN_2_
*M* *_r_* = 400.93
Monoclinic, 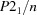

*a* = 10.471 (2) Å
*b* = 9.7798 (19) Å
*c* = 21.682 (4) Å
β = 91.080 (4)°
*V* = 2219.9 (7) Å^3^
*Z* = 4
Mo *K*α radiation
μ = 0.19 mm^−1^
*T* = 150 K
0.36 × 0.14 × 0.11 mm

#### Data collection

Bruker APEX 2000 CCD area-detector diffractometer
Absorption correction: multi-scan (*SADABS*; Bruker, 2001 ▶) *T*_min_ = 0.969, *T*_max_ = 0.980
16940 measured reflections
4346 independent reflections
2593 reflections with *I* > 2σ(*I*)
*R*_int_ = 0.088

#### Refinement

*R*[*F* ^2^ > 2σ(*F* ^2^)] = 0.053
*wR*(*F* ^2^) = 0.110
*S* = 0.89
4346 reflections
263 parameters
H-atom parameters constrained
Δρ_max_ = 0.26 e Å^−3^
Δρ_min_ = −0.31 e Å^−3^

### 

Data collection: *SMART* (Bruker, 2001 ▶); cell refinement: *SAINT* (Bruker, 2001 ▶); data reduction: *SAINT*; program(s) used to solve structure: *SHELXS97* (Sheldrick, 2008 ▶); program(s) used to refine structure: *SHELXL97* (Sheldrick, 2008 ▶); molecular graphics: *ORTEP-3 for Windows* (Farrugia, 2012 ▶); software used to prepare material for publication: *WinGX* (Farrugia, 2012 ▶) and *PLATON* (Spek, 2009 ▶).

## Supplementary Material

Crystal structure: contains datablock(s) global, I. DOI: 10.1107/S1600536813018229/yk2095sup1.cif

Structure factors: contains datablock(s) I. DOI: 10.1107/S1600536813018229/yk2095Isup2.hkl

Additional supplementary materials:  crystallographic information; 3D view; checkCIF report

## Figures and Tables

**Table 1:** Hydrogen-bond geometry (Å, °)

*D*—H⋯*A*	*D*—H	H⋯*A*	*D*⋯*A*	*D*—H⋯*A*
C20—H20⋯C25^i^	0.95	3.00	3.887 (3)	157
C24—H24⋯C13^ii^	0.95	2.78	3.697 (3)	163
C28—H28⋯N3^iii^	0.95	2.88	3.444 (3)	119
C28—H28⋯C17^iii^	0.95	3.00	3.894 (3)	158

Symmetry codes: (i) 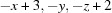; (ii) 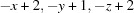; (iii) 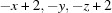.

**Fig. 1.**
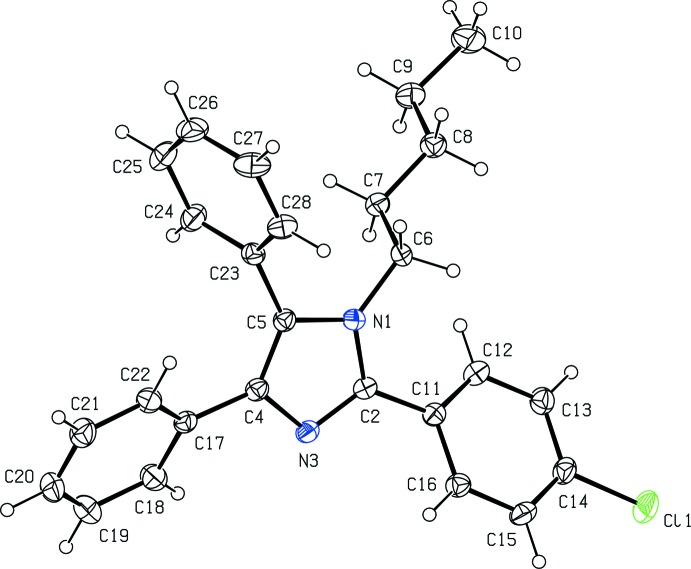
The structure of the title molecule with the atom numbering scheme. Displacement ellipsoids for non-H atoms are drawn at the 30% probability level.

**Fig. 2.**
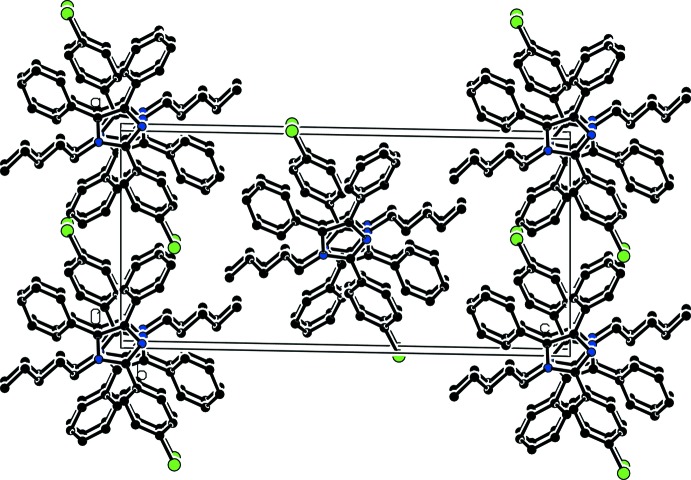
View of the packing diagram of the title compound along the b axis direction. All H atoms omitted for clarity.

## supplementary crystallographic information

### Comment

Various substituted imidazole derivatives have been found to possess a significant biological exhibition such as anti-helminthic, analgesic, antibacterial, antifungal, antiviral, tuberculostatic, cytostatic, and anti-inflammation activities (Shalini *et al.*, 2011). Tetra-substituted imidazoles in particular represent the core structure in many biological systems such as Losartan, Trifenagrel, Eprosartan and Olmesartan (Ramesh *et al.*, 2012; Wolkenberg *et al.*, 2004). Based on literature and further to our ongoing study in synthesis of bio-active molecules the title compound has been synthesized and herein we report its crystal structure.

As shown in Fig. 1, the title molecule adopts a non-planar conformation. The two phenyl (C17–C22 and C23–C28) and 2-(4-chlorophenyl) (C11–C16) groups make the dihedral angles of 30.03 (11), 67.49 (12) and 41.56 (11) °, respectively, with the imidazole ring (N1/C2/N3/C4/C5). The N1–C6–C7–C8 and C7–C8–C9–C10 torsion angles are -168.61 (16) and -176.82 (19)°, respectively. The values of the geometric parameters of (I) are in the normal range and comparable to those reported for the similar compounds (Simpson *et al.*, 2013; Akkurt *et al.*, 2013).

The crystal structure of (I) is stabilized by intra and intermolecular Cl···H(C) contacts [Cl1···H13 = 2.80 Å, Cl1···H15 = 2.80 Å, Cl1···H6A*^i^* = 3.10 Å, Cl1···H8B*^i^* = 3.05 Å, Cl1···H24*^ii^* = 3.14 Å, Cl1···H10B*^iii^* = 3.02 Å (symmetry codes: (*i*) 1 - *x*, 1 - *y*, 2 - *z*; (*ii*) 2 - *x*, 1 - *y*, 2 - *z*; (*iii*) -1/2 + *x*, 3/2 - *y*, 1/2 + *z*)]. Fig. 2 shows the molecular packing of (I) along the *b* axis.

### Experimental

The title compound was synthesized following the previously reported procedure (Simpson *et al.*, 2013). Colourless grains of product (*M*.p. 399 K) were collected with 89% yield. The crystals of sufficient quality for X-ray diffraction study were obtained by slow evaporation of the ethanol solution of the title compound.

### Refinement

All H atoms were placed in geometrically idealized positions [C—H = 0.95 Å (aromatic), C—H = 0.99 Å (methylene), and C—H = 0.98 Å (methyl)] and refined as riding on their parent atoms with *U*_iso_(H) = 1.2 *U*_eq_ (C). treated as riding on their parent atoms, with *U*_iso_(H) = 1.2–1.5*U*_eq_(C).

### Crystal data

**Table:**

C_26_H_25_ClN_2_	*F*(000) = 848
*M**_r_* = 400.93	*D*_x_ = 1.200 Mg m^−^^3^
Monoclinic, *P*2_1_/*n*	Mo *K*α radiation, λ = 0.71073 Å
Hall symbol: -P 2yn	Cell parameters from 811 reflections
*a* = 10.471 (2) Å	θ = 2.8–23.3°
*b* = 9.7798 (19) Å	µ = 0.19 mm^−^^1^
*c* = 21.682 (4) Å	*T* = 150 K
β = 91.080 (4)°	Block, colourless
*V* = 2219.9 (7) Å^3^	0.36 × 0.14 × 0.11 mm
*Z* = 4	

### Data collection

**Table:**

Bruker APEX 2000 CCD area-detector diffractometer	4346 independent reflections
Radiation source: fine-focus sealed tube	2593 reflections with *I* > 2σ(*I*)
Graphite monochromator	*R*_int_ = 0.088
φ and ω scans	θ_max_ = 26.0°, θ_min_ = 1.9°
Absorption correction: multi-scan (*SADABS*; Bruker, 2001)	*h* = −12→12
*T*_min_ = 0.969, *T*_max_ = 0.980	*k* = −12→11
16940 measured reflections	*l* = −26→26

### Refinement

**Table:**

Refinement on *F*^2^	0 restraints
Least-squares matrix: full	Hydrogen site location: inferred from neighbouring sites
*R*[*F*^2^ > 2σ(*F*^2^)] = 0.053	H-atom parameters constrained
*wR*(*F*^2^) = 0.110	W = 1/[Σ^2^(*F*O^2^) + (0.0377*P*)^2^] WHERE *P* = (*F*O^2^ + 2*F*C^2^)/3
*S* = 0.89	(Δ/σ)_max_ = 0.001
4346 reflections	Δρ_max_ = 0.26 e Å^−^^3^
263 parameters	Δρ_min_ = −0.31 e Å^−^^3^

### Special details

**Table:**

Geometry. Bond distances, angles *etc*. have been calculated using the rounded fractional coordinates. All s.u.'s are estimated from the variances of the (full) variance-covariance matrix. The cell s.u.'s are taken into account in the estimation of distances, angles and torsion angles
Refinement. Refinement on *F*^2^ for ALL reflections except those flagged by the user for potential systematic errors. Weighted *R*-factors *wR* and all goodnesses of fit *S* are based on *F*^2^, conventional *R*-factors *R* are based on *F*, with *F* set to zero for negative *F*^2^. The observed criterion of *F*^2^ > σ(*F*^2^) is used only for calculating -*R*-factor-obs *etc*. and is not relevant to the choice of reflections for refinement. *R*-factors based on *F*^2^ are statistically about twice as large as those based on *F*, and *R*-factors based on ALL data will be even larger.

### Fractional atomic coordinates and isotropic or equivalent isotropic displacement parameters (Å^2^)

**Table:**

	*x*	*y*	*z*	*U*_iso_*/*U*_eq_	
Cl1	0.48467 (6)	0.57406 (7)	1.11900 (3)	0.0565 (3)	
N1	0.95325 (15)	0.25818 (17)	0.95080 (7)	0.0286 (6)	
N3	1.02899 (16)	0.24351 (18)	1.04691 (7)	0.0308 (6)	
C2	0.9371 (2)	0.2951 (2)	1.01115 (9)	0.0286 (7)	
C4	1.10784 (19)	0.1722 (2)	1.00835 (9)	0.0295 (7)	
C5	1.06275 (19)	0.1789 (2)	0.94862 (9)	0.0288 (7)	
C6	0.87851 (19)	0.3032 (2)	0.89685 (9)	0.0304 (7)	
C7	0.94069 (19)	0.4250 (2)	0.86571 (9)	0.0338 (8)	
C8	0.8550 (2)	0.4923 (2)	0.81788 (10)	0.0373 (8)	
C9	0.9151 (2)	0.6130 (3)	0.78703 (11)	0.0500 (9)	
C10	0.8260 (2)	0.6845 (3)	0.74173 (11)	0.0629 (11)	
C11	0.82661 (19)	0.3684 (2)	1.03559 (9)	0.0293 (7)	
C12	0.7711 (2)	0.4823 (2)	1.00771 (10)	0.0358 (8)	
C13	0.6648 (2)	0.5448 (2)	1.03269 (10)	0.0393 (8)	
C14	0.6155 (2)	0.4937 (3)	1.08642 (10)	0.0384 (8)	
C15	0.6700 (2)	0.3823 (2)	1.11571 (10)	0.0381 (8)	
C16	0.7750 (2)	0.3207 (2)	1.09032 (9)	0.0336 (8)	
C17	1.22136 (19)	0.1012 (2)	1.03386 (9)	0.0304 (7)	
C18	1.2830 (2)	0.1515 (3)	1.08647 (10)	0.0429 (9)	
C19	1.3879 (2)	0.0841 (3)	1.11173 (11)	0.0534 (10)	
C20	1.4320 (2)	−0.0343 (3)	1.08535 (12)	0.0519 (10)	
C21	1.3724 (2)	−0.0846 (3)	1.03348 (11)	0.0462 (9)	
C22	1.2677 (2)	−0.0180 (2)	1.00781 (10)	0.0366 (8)	
C23	1.1111 (2)	0.1239 (2)	0.88975 (9)	0.0304 (8)	
C24	1.2243 (2)	0.1728 (3)	0.86639 (10)	0.0445 (9)	
C25	1.2713 (2)	0.1218 (3)	0.81166 (11)	0.0564 (10)	
C26	1.2062 (3)	0.0227 (3)	0.77984 (11)	0.0522 (10)	
C27	1.0928 (3)	−0.0263 (3)	0.80242 (10)	0.0509 (10)	
C28	1.0457 (2)	0.0242 (2)	0.85709 (10)	0.0401 (8)	
H6A	0.79150	0.32870	0.90970	0.0360*	
H6B	0.87070	0.22690	0.86700	0.0360*	
H7A	0.96480	0.49340	0.89750	0.0410*	
H7B	1.02000	0.39420	0.84580	0.0410*	
H8A	0.83120	0.42380	0.78600	0.0450*	
H8B	0.77550	0.52250	0.83790	0.0450*	
H9A	0.99180	0.58190	0.76500	0.0600*	
H9B	0.94330	0.67920	0.81900	0.0600*	
H10A	0.79660	0.61930	0.71020	0.0950*	
H10B	0.87130	0.75990	0.72200	0.0950*	
H10C	0.75220	0.72050	0.76360	0.0950*	
H12	0.80650	0.51800	0.97100	0.0430*	
H13	0.62660	0.62170	1.01300	0.0470*	
H15	0.63550	0.34850	1.15290	0.0460*	
H16	0.81290	0.24420	1.11050	0.0400*	
H18	1.25280	0.23280	1.10530	0.0510*	
H19	1.42970	0.11990	1.14750	0.0640*	
H20	1.50340	−0.08080	1.10310	0.0620*	
H21	1.40310	−0.16590	1.01500	0.0550*	
H22	1.22700	−0.05430	0.97190	0.0440*	
H24	1.27040	0.24200	0.88810	0.0530*	
H25	1.34940	0.15600	0.79620	0.0680*	
H26	1.23870	−0.01230	0.74240	0.0630*	
H27	1.04690	−0.09500	0.78040	0.0610*	
H28	0.96750	−0.01010	0.87230	0.0480*	

### Atomic displacement parameters (Å^2^)

**Table:**

	*U*^11^	*U*^22^	*U*^33^	*U*^12^	*U*^13^	*U*^23^
Cl1	0.0404 (4)	0.0733 (5)	0.0562 (4)	0.0153 (3)	0.0123 (3)	−0.0120 (4)
N1	0.0299 (10)	0.0311 (11)	0.0249 (10)	0.0013 (9)	0.0005 (8)	0.0005 (8)
N3	0.0310 (10)	0.0337 (12)	0.0279 (10)	0.0011 (9)	0.0048 (8)	−0.0006 (9)
C2	0.0315 (13)	0.0272 (13)	0.0273 (12)	−0.0010 (10)	0.0035 (10)	−0.0005 (10)
C4	0.0312 (12)	0.0274 (13)	0.0301 (12)	0.0002 (10)	0.0042 (10)	0.0013 (10)
C5	0.0292 (12)	0.0275 (13)	0.0298 (12)	0.0013 (10)	0.0029 (10)	0.0007 (10)
C6	0.0263 (12)	0.0357 (14)	0.0290 (12)	0.0025 (10)	−0.0025 (10)	−0.0003 (10)
C7	0.0326 (13)	0.0381 (15)	0.0309 (12)	−0.0019 (11)	0.0025 (10)	0.0019 (11)
C8	0.0393 (14)	0.0359 (15)	0.0366 (13)	−0.0003 (11)	0.0021 (11)	0.0027 (11)
C9	0.0488 (15)	0.0581 (19)	0.0432 (15)	0.0014 (14)	0.0070 (12)	0.0190 (14)
C10	0.071 (2)	0.063 (2)	0.0550 (18)	0.0116 (16)	0.0071 (15)	0.0256 (15)
C11	0.0298 (12)	0.0291 (14)	0.0291 (12)	−0.0025 (10)	0.0013 (10)	−0.0034 (10)
C12	0.0378 (14)	0.0355 (15)	0.0344 (13)	−0.0015 (11)	0.0081 (11)	−0.0004 (11)
C13	0.0353 (14)	0.0414 (16)	0.0412 (14)	0.0057 (11)	0.0010 (11)	−0.0024 (12)
C14	0.0317 (13)	0.0453 (16)	0.0383 (14)	0.0041 (12)	0.0053 (11)	−0.0108 (12)
C15	0.0401 (14)	0.0449 (16)	0.0295 (13)	−0.0035 (12)	0.0099 (11)	−0.0039 (12)
C16	0.0365 (13)	0.0327 (14)	0.0317 (13)	0.0019 (11)	0.0028 (11)	−0.0014 (11)
C17	0.0300 (12)	0.0316 (14)	0.0299 (12)	−0.0008 (11)	0.0056 (10)	0.0064 (11)
C18	0.0434 (15)	0.0466 (17)	0.0385 (14)	0.0065 (12)	−0.0032 (12)	−0.0010 (12)
C19	0.0455 (16)	0.070 (2)	0.0441 (15)	0.0057 (15)	−0.0117 (13)	0.0017 (15)
C20	0.0370 (15)	0.065 (2)	0.0537 (17)	0.0133 (14)	−0.0014 (13)	0.0149 (15)
C21	0.0429 (15)	0.0426 (17)	0.0533 (16)	0.0119 (13)	0.0082 (13)	0.0090 (14)
C22	0.0373 (14)	0.0348 (15)	0.0378 (13)	0.0022 (11)	0.0018 (11)	0.0057 (11)
C23	0.0344 (13)	0.0294 (14)	0.0275 (12)	0.0072 (11)	0.0003 (10)	0.0017 (10)
C24	0.0357 (14)	0.0588 (18)	0.0391 (14)	−0.0044 (12)	0.0073 (11)	−0.0100 (13)
C25	0.0418 (15)	0.082 (2)	0.0458 (16)	0.0031 (15)	0.0155 (13)	−0.0067 (16)
C26	0.0646 (19)	0.062 (2)	0.0304 (14)	0.0183 (15)	0.0098 (13)	−0.0049 (14)
C27	0.078 (2)	0.0425 (17)	0.0323 (14)	−0.0033 (15)	0.0013 (13)	−0.0064 (12)
C28	0.0503 (15)	0.0360 (15)	0.0341 (13)	−0.0045 (12)	0.0049 (12)	0.0016 (11)

### Geometric parameters (Å, º)

**Table:**

Cl1—C14	1.741 (2)	C25—C26	1.365 (4)
N1—C2	1.371 (2)	C26—C27	1.379 (4)
N1—C5	1.386 (3)	C27—C28	1.384 (3)
N1—C6	1.463 (3)	C6—H6A	0.9900
N3—C2	1.324 (3)	C6—H6B	0.9900
N3—C4	1.376 (3)	C7—H7A	0.9900
C2—C11	1.468 (3)	C7—H7B	0.9900
C4—C5	1.372 (3)	C8—H8A	0.9900
C4—C17	1.475 (3)	C8—H8B	0.9900
C5—C23	1.483 (3)	C9—H9A	0.9900
C6—C7	1.522 (3)	C9—H9B	0.9900
C7—C8	1.510 (3)	C10—H10A	0.9800
C8—C9	1.501 (3)	C10—H10B	0.9800
C9—C10	1.513 (3)	C10—H10C	0.9800
C11—C12	1.390 (3)	C12—H12	0.9500
C11—C16	1.394 (3)	C13—H13	0.9500
C12—C13	1.389 (3)	C15—H15	0.9500
C13—C14	1.377 (3)	C16—H16	0.9500
C14—C15	1.379 (3)	C18—H18	0.9500
C15—C16	1.378 (3)	C19—H19	0.9500
C17—C18	1.390 (3)	C20—H20	0.9500
C17—C22	1.387 (3)	C21—H21	0.9500
C18—C19	1.385 (3)	C22—H22	0.9500
C19—C20	1.375 (4)	C24—H24	0.9500
C20—C21	1.367 (4)	C25—H25	0.9500
C21—C22	1.383 (3)	C26—H26	0.9500
C23—C24	1.383 (3)	C27—H27	0.9500
C23—C28	1.379 (3)	C28—H28	0.9500
C24—C25	1.386 (3)		
			
C2—N1—C5	107.28 (16)	C6—C7—H7B	109.00
C2—N1—C6	127.60 (16)	C8—C7—H7A	109.00
C5—N1—C6	124.87 (16)	C8—C7—H7B	109.00
C2—N3—C4	105.97 (16)	H7A—C7—H7B	108.00
N1—C2—N3	110.93 (17)	C7—C8—H8A	109.00
N1—C2—C11	125.82 (18)	C7—C8—H8B	109.00
N3—C2—C11	122.92 (17)	C9—C8—H8A	109.00
N3—C4—C5	110.47 (17)	C9—C8—H8B	109.00
N3—C4—C17	119.95 (17)	H8A—C8—H8B	108.00
C5—C4—C17	129.58 (18)	C8—C9—H9A	109.00
N1—C5—C4	105.36 (17)	C8—C9—H9B	109.00
N1—C5—C23	121.90 (17)	C10—C9—H9A	109.00
C4—C5—C23	132.71 (19)	C10—C9—H9B	109.00
N1—C6—C7	111.31 (16)	H9A—C9—H9B	108.00
C6—C7—C8	113.10 (16)	C9—C10—H10A	109.00
C7—C8—C9	113.60 (17)	C9—C10—H10B	109.00
C8—C9—C10	113.21 (19)	C9—C10—H10C	110.00
C2—C11—C12	124.08 (18)	H10A—C10—H10B	109.00
C2—C11—C16	117.69 (18)	H10A—C10—H10C	109.00
C12—C11—C16	118.23 (18)	H10B—C10—H10C	109.00
C11—C12—C13	120.99 (19)	C11—C12—H12	119.00
C12—C13—C14	119.1 (2)	C13—C12—H12	120.00
Cl1—C14—C13	119.34 (19)	C12—C13—H13	120.00
Cl1—C14—C15	119.46 (17)	C14—C13—H13	120.00
C13—C14—C15	121.2 (2)	C14—C15—H15	120.00
C14—C15—C16	119.20 (19)	C16—C15—H15	120.00
C11—C16—C15	121.28 (19)	C11—C16—H16	119.00
C4—C17—C18	119.97 (19)	C15—C16—H16	119.00
C4—C17—C22	121.91 (18)	C17—C18—H18	120.00
C18—C17—C22	118.1 (2)	C19—C18—H18	120.00
C17—C18—C19	120.6 (2)	C18—C19—H19	120.00
C18—C19—C20	120.4 (2)	C20—C19—H19	120.00
C19—C20—C21	119.6 (2)	C19—C20—H20	120.00
C20—C21—C22	120.5 (2)	C21—C20—H20	120.00
C17—C22—C21	120.8 (2)	C20—C21—H21	120.00
C5—C23—C24	119.91 (19)	C22—C21—H21	120.00
C5—C23—C28	121.60 (19)	C17—C22—H22	120.00
C24—C23—C28	118.5 (2)	C21—C22—H22	120.00
C23—C24—C25	120.6 (2)	C23—C24—H24	120.00
C24—C25—C26	120.4 (2)	C25—C24—H24	120.00
C25—C26—C27	119.5 (2)	C24—C25—H25	120.00
C26—C27—C28	120.2 (2)	C26—C25—H25	120.00
C23—C28—C27	120.7 (2)	C25—C26—H26	120.00
N1—C6—H6A	109.00	C27—C26—H26	120.00
N1—C6—H6B	109.00	C26—C27—H27	120.00
C7—C6—H6A	109.00	C28—C27—H27	120.00
C7—C6—H6B	109.00	C23—C28—H28	120.00
H6A—C6—H6B	108.00	C27—C28—H28	120.00
C6—C7—H7A	109.00		
			
C5—N1—C2—N3	−0.3 (2)	N1—C6—C7—C8	−168.61 (16)
C5—N1—C2—C11	−173.78 (19)	C6—C7—C8—C9	179.75 (18)
C6—N1—C2—N3	−174.66 (18)	C7—C8—C9—C10	−176.82 (19)
C6—N1—C2—C11	11.9 (3)	C2—C11—C12—C13	178.54 (19)
C2—N1—C5—C4	−0.2 (2)	C16—C11—C12—C13	−1.8 (3)
C2—N1—C5—C23	−178.41 (18)	C2—C11—C16—C15	−178.87 (19)
C6—N1—C5—C4	174.38 (17)	C12—C11—C16—C15	1.4 (3)
C6—N1—C5—C23	−3.9 (3)	C11—C12—C13—C14	1.0 (3)
C2—N1—C6—C7	94.4 (2)	C12—C13—C14—Cl1	178.44 (17)
C5—N1—C6—C7	−79.0 (2)	C12—C13—C14—C15	0.2 (3)
C4—N3—C2—N1	0.7 (2)	Cl1—C14—C15—C16	−178.79 (17)
C4—N3—C2—C11	174.34 (18)	C13—C14—C15—C16	−0.5 (3)
C2—N3—C4—C5	−0.8 (2)	C14—C15—C16—C11	−0.3 (3)
C2—N3—C4—C17	179.98 (19)	C4—C17—C18—C19	178.5 (2)
N1—C2—C11—C12	−45.1 (3)	C22—C17—C18—C19	0.2 (3)
N1—C2—C11—C16	135.2 (2)	C4—C17—C22—C21	−178.4 (2)
N3—C2—C11—C12	142.2 (2)	C18—C17—C22—C21	−0.1 (3)
N3—C2—C11—C16	−37.5 (3)	C17—C18—C19—C20	−0.5 (4)
N3—C4—C5—N1	0.6 (2)	C18—C19—C20—C21	0.7 (4)
N3—C4—C5—C23	178.6 (2)	C19—C20—C21—C22	−0.6 (4)
C17—C4—C5—N1	179.72 (19)	C20—C21—C22—C17	0.3 (4)
C17—C4—C5—C23	−2.3 (4)	C5—C23—C24—C25	179.5 (2)
N3—C4—C17—C18	−29.6 (3)	C28—C23—C24—C25	−0.5 (3)
N3—C4—C17—C22	148.7 (2)	C5—C23—C28—C27	−179.6 (2)
C5—C4—C17—C18	151.4 (2)	C24—C23—C28—C27	0.4 (3)
C5—C4—C17—C22	−30.4 (3)	C23—C24—C25—C26	0.2 (4)
N1—C5—C23—C24	111.6 (2)	C24—C25—C26—C27	0.2 (4)
N1—C5—C23—C28	−68.3 (3)	C25—C26—C27—C28	−0.3 (4)
C4—C5—C23—C24	−66.1 (3)	C26—C27—C28—C23	0.0 (4)
C4—C5—C23—C28	114.0 (3)		

### Hydrogen-bond geometry (Å, º)

**Table:**

*D*—H···*A*	*D*—H	H···*A*	*D*···*A*	*D*—H···*A*
C20—H20···C25^i^	0.95	3.00	3.887 (3)	157
C24—H24···C13^ii^	0.95	2.78	3.697 (3)	163
C28—H28···N3^iii^	0.95	2.88	3.444 (3)	119
C28—H28···C17^iii^	0.95	3.00	3.894 (3)	158

Symmetry codes: (i) −*x*+3, −*y*, −*z*+2; (ii) −*x*+2, −*y*+1, −*z*+2; (iii) −*x*+2, −*y*, −*z*+2.
